# Engineering bacteriocin‐mediated resistance against the plant pathogen *Pseudomonas syringae*

**DOI:** 10.1111/pbi.13294

**Published:** 2019-12-03

**Authors:** William M. Rooney, Rhys W. Grinter, Annapaula Correia, Julian Parkhill, Daniel C. Walker, Joel J. Milner

**Affiliations:** ^1^ Plant Science Group Institute of Molecular, Cell and Systems Biology & School of Life Sciences University of Glasgow Glasgow UK; ^2^ Institute of Infection, Immunity & Inflammation College of Medical, Veterinary & Life Sciences University of Glasgow Glasgow UK; ^3^ Wellcome Trust Sanger Institute Wellcome Trust Genome Campus Hinxton UK; ^4^ Present address: School of Biological Sciences Centre for Geometric Biology Monash University Clayton Victoria 3800 Australia; ^5^ Present address: Department of Zoology University of Oxford South Parks Road Oxford OX1 3PS UK; ^6^ Present address: Department of Veterinary Medicine University of Cambridge Madingley Road Cambridge CB3 0ES UK

**Keywords:** putidacin L1, Arabidopsis, tobacco, bacteriocins, transgenic, disease resistance antimicrobial peptide

## Abstract

The plant pathogen, *Pseudomonas syringae* (*Ps*), together with related *Ps* species, infects and attacks a wide range of agronomically important crops, including tomato, kiwifruit, pepper, olive and soybean, causing economic losses. Currently, chemicals and introduced resistance genes are used to protect plants against these pathogens but have limited success and may have adverse environmental impacts. Consequently, there is a pressing need to develop alternative strategies to combat bacterial disease in crops. One such strategy involves using narrow‐spectrum protein antibiotics (so‐called bacteriocins), which diverse bacteria use to compete against closely related species. Here, we demonstrate that one bacteriocin, putidacin L1 (PL1), can be expressed in an active form at high levels in Arabidopsis and in *Nicotiana benthamiana in planta* to provide effective resistance against diverse pathovars of *Ps.* Furthermore, we find that *Ps* strains that mutate to acquire tolerance to PL1 lose their O‐antigen, exhibit reduced motility and still cannot induce disease symptoms in PL1‐transgenic Arabidopsis. Our results provide proof‐of‐principle that the transgene‐mediated expression of a bacteriocin *in planta* can provide effective disease resistance to bacterial pathogens. Thus, the expression of bacteriocins in crops might offer an effective strategy for managing bacterial disease, in the same way that the genetic modification of crops to express insecticidal proteins has proven to be an extremely successful strategy for pest management. Crucially, nearly all genera of bacteria, including many plant pathogenic species, produce bacteriocins, providing an extensive source of these antimicrobial agents.

## Introduction

*Pseudomonas syringae* (*Ps*) is a Gram‐negative bacterial plant pathogen. The *Ps* species complex consists of over 50 known pathovars (pv.), which are responsible for a variety of different diseases, such as spot and blight disease and bacterial speck, in a wide range of agronomically important crops, including tomato, beans and tobacco (O’Brien *et al.*, 2011; Lamichhane *et al.*, 2014; Lamichhane *et al.*, 2015). Once a plant pathogen is introduced into a crop, it can spread rapidly because of the lack of genetic diversity in commercial crop varieties (Esquinas‐Alcázar, 2005).

A recent example of this is the pandemic caused by *Ps* pv. *actinidiae* (*Psa*), which is currently causing great damage to the global kiwifruit industry (Vanneste, 2017). The emergence of canker disease on commercial kiwifruit (*Actinidia *spp.) varieties has been well documented since the early years of *A. deliciosa* domestication in Japan in 1984 (Serizawa *et al.*, 1989) and has subsequently spread worldwide (Takikawa *et al.*, 1989; Scortichini, 1994), and the emergence of hypervirulent strains of *Psa* has exacerbated the problem (Balestra *et al.*, 2009; Everett *et al.*, 2011). For example, *Psa* was detected in 37% of New Zealand’s kiwifruit orchards, with the total cost to the industry perhaps exceeding $1.33 billion (Vanneste, 2017).

Currently, chemicals (e.g. copper salts or antibiotics) are used to protect crops from these bacterial pathogens, often with limited success. They also may have adverse environmental impacts because of off‐target activity and can encourage the evolution of resistance among bacterial populations (Damalas and Eleftherohorinos, 2011; Sundin and Bender, 1993). The introduction of resistance genes, such as *EFR* in tobacco and tomato, has been successful in providing resistance against *Ps* (Lacombe *et al.*, 2010)*.* However, there is a distinct lack of diversity of suitable natural resistance genes that can be introduced into commercial crops. There is therefore a pressing need to develop new technologies to introduce disease resistance into economically important crops to protect them from plant pathogens like *Ps*.

The large *Ps* species complex means that individual *Ps* species are under intense selective pressure to evolve mechanisms to eliminate inter‐ and intra‐species competition in their environmental niche. One mechanism used to eliminate competitor strains is the production of bacteriocins, which are narrow‐spectrum, proteinaceous antibiotics that target and kill related bacterial species. The highly targeted, antibiotic activity of bacteriocins could potentially be exploited to provide crops with protection against specific bacterial pathogens with minimal impact on the wider microbial community (Riley and Wertz, 2002).

Various prospective bacteriocins have been identified in *Pseudomonas *spp., including putidacin L1 (PL1), a 30 kDa lectin‐like bacteriocin that is highly potent against *Ps* pv. *syringae* ***,*** *lachrymans* and *morsprunorum* (Parret *et al.*, 2003; Parret *et al.*, 2005). The lectin‐like bacteriocins bind to D‐rhamnose‐containing oligosaccharides that are incorporated into lipopolysaccharide (LPS) on the bacterial surface (Ghequire *et al.*, 2013; McCaughey *et al.*, 2014). This binding facilitates the docking of PL1 on the cell surface and its interaction with the outer membrane insertase BamA, leading to the death of the cell via an unknown mechanism (Ghequire *et al.*, 2018). We are not aware of any prior reports of attempts to express bacteriocins *in planta* as a strategy to confer resistance against plant pathogenic bacteria. Bacteriocins with activities against *E. coli*, *Salmonella* and *Pseudomonas aeruginosa* have been expressed in plants but with the objective of using these as a means of treating bacterial infections in humans (Schulz *et al.*, 2015; Paškevičius *et al.*, 2017; Schneider *et al.*, 2018). The resulting successful demonstration that active bacteriocins can be expressed *in planta* suggests that PL1 could also be expressed *in planta* in an active form to protect plants against *Ps* infection.

The use of novel peptides such as antimicrobial peptides (AMPs) for defence against pathogens in agriculture is not a novel concept and there are a number of reports of AMPs being tested as a strategy for conferring pathogen resistance (De Souza Cândido *et al.*, 2014; Holaskova *et al.*, 2014; Ageitos *et al.*, 2017). Interestingly, AMP activity is not always mirrored *in planta* compared with activity *in vitro*. This is mainly attributable to factors such as salt concentration, protease‐based degradation and inhibition by phenolic compounds (Zeitler *et al.*, 2013). Furthermore, AMPs have been shown to be sensitive to divalent cations common in the apoplastic fluid‐like Ca^2+^ and Mg^2+^, which can drastically reduce their efficacy (De Bolle *et al.*, 1996; Güell *et al.*, 2011). Therefore, bacteriocins exhibit significant potential advantages over more generalized antimicrobials because of their highly targeted activity at low concentrations.

In this study, we explore this possibility and demonstrate that active PL1 can be efficiently expressed in both *Nicotiana benthamiana* and Arabidopsis. We show that the transient expression of PL1 in *N. benthamiana* and its stable expression in Arabidopsis provides quantitative and qualitative disease resistance against PL1‐sensitive strains of *Ps*. Furthermore, we show that mutations associated with PL1‐insensitivity/tolerance are linked to the LPS biosynthesis machinery and that *Ps* mutants with increased tolerance to PL1 are still unable to induce disease symptoms in transgenic plants. We conclude from our results that the transgenic expression of a bacteriocin *in planta* can provide robust disease resistance against the bacterial phytopathogen *Ps*.

## Results

### PL1 has a narrow killing spectrum

To determine the killing spectrum of PL1 against *Ps* pathovars, recombinant PL1‐His_6_ was purified from *E. coli.* The killing activity of the purified protein was then assessed against a panel of 22 diverse *Ps* pathovars, including pathogens of kiwifruit, locust bean, oat, soybean, cucumber, cabbage, cherry, plum, olive, pear, maize, lilac and tomato. Of the 22 strains tested, 10 (from 6 different *Ps* pathovars) were sensitive to PL1 (Table 1 and Figure S1), including all three members of the *syringae* group. Minimum inhibitory concentrations of PL1 ranged from 0.85 nm to 1.8 µm, with several of the pathovars showing sensitivity at nanomolar concentrations. All 4 members of the tomato group were resistant to PL1. We conclude that PL1 has a very specific killing spectrum making it an ideal candidate for expression in plants.

**Table 1:** The range of P. syringae pathovars that are susceptible to PL1

Pathovar	Strain ID	Sensitive to 10 µm PL1?	MIC (nM)	Origin	Host
*actinidae*	NCPPB 3738	Yes	125	Japan	*Actinidiae delici*
*actinidae*	NCPPB 3739	Yes	125	Japan	*Actinidiae delici*
*ciccaronei*	NCPPB 2355	No	–	Italy	*Ceratonia siliqua*
*coronafaciens*	LMG 5060	No	–	UK	*Avena sativa*
*glycinea*	NCPPB 2070	Yes	1070	USA	*Glycine max*
*glycinea*	NCPPB 1245	Yes	1070	Canada	*Glycine max*
*glycinea*	NCPPB 2895	No	–	Australia	*Glycine max*
*glycinea*	NCPPB 3643	No	–	Brazil	*Glycine max*
*lachrymans*	LMG 5456	Yes	22.9	UK	*Cucumis sativus*
*maculicola*	LMG 2208	No	–	UK	*Brassica oleraccea*
*morsprunorum*	LMG 2222	Yes	0.85	UK	*Prunus avium*
*persicae*	NCPPB 3687	No	–	New Zealand	*Prunus salicina*
*persicae*	NCPPB 2254	No	–	France	*Prunus salicina*
*savastoni*	NCPPB 1506	Yes	325	Italy	*Olea europaea*
*savastoni*	NCPPB 2327	No	–	Italy	*Olea europaea*
*syringae*	LMG 5084	Yes	5.6	UK	*Pyrus communis*
*syringae*	LMG 5082	Yes	8.3	UK	*Zea Mays*
*syringae*	LMG 1247	Yes	1850	UK	*Syringa vulgaris*
*tomato*	NCPPB 3160	No	–	UK	*Solanum lycopersicum*
*tomato*	NCPPB 2563	No	–	UK	*Solanum lycopersicum*
*tomato*	NCPPB 1107	No	–	UK	*Solanum lycopersicum*
*tomato*	DC3000	No	–	USA	*Solanum lycopersicum*

### Expression of PL1 in *N. benthamiana* provides robust resistance against *Ps* pv. *syringae* LMG5084

**Figure 1:**
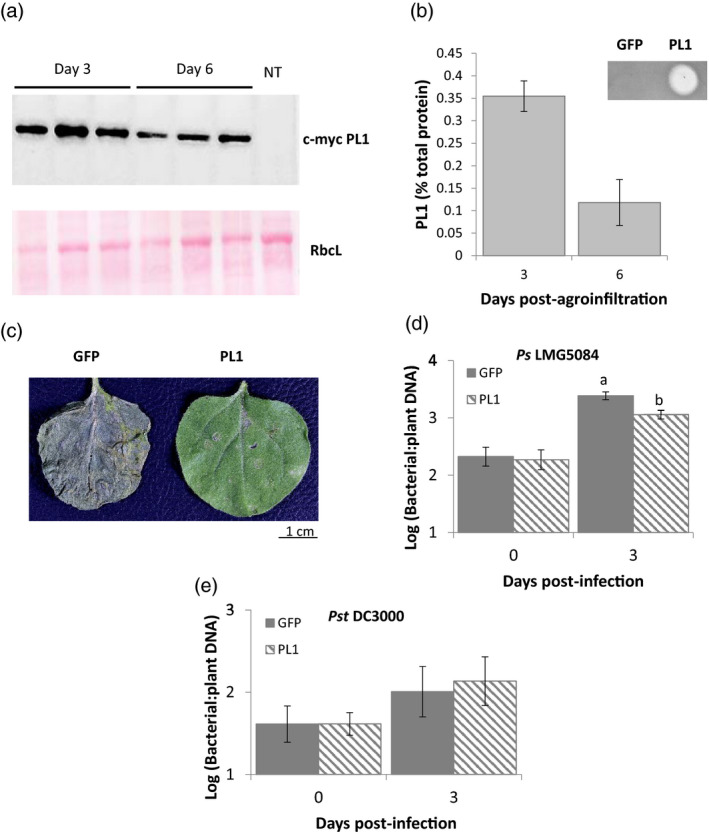
Transient expression of PL1 in *N. benthamiana* attenuates the growth of PL1‐susceptible strains but not of PL1‐resistant strains. a, PL1 can be expressed at high levels in *N. benthamiana*. (Upper) Western blot of myc‐tagged PL1 expressed in *N. benthamiana* leaves, 3‐ and 6‐day post‐infiltration; (lower) the corresponding Ponceau S staining of the large RuBisCO subunit (rbcL) was used as a loading control and with a non‐transgenic control (NT). b, % Total PL1 protein and activity from transient PL1 expression. Serial dilutions of whole‐protein extracts from *N. benthamiana* leaves, 3‐ and 6‐day post‐infiltration, were spotted onto lawns of *Ps* LMG5084 to estimate the percentage of PL1 protein in from the total plant protein extracts. The top right panel shows a lawn of *Ps* LMG5084 with spots of *N. benthamiana* extracts, expressing either PL1 or GFP (control). Error bars represent the standard deviation of 3 independent replicates. c, PL1‐expressing leaves showed qualitative resistance to *Ps* LMG5084. *N. Benthamiana* leaves that express either PL1 or GFP were infiltrated with *Ps* LMG5084 (3‐day post‐agroinfiltration) and were left for 7 days post‐infection for symptoms to develop. d, PL1‐expressing leaves show quantitative resistance to *Ps* LMG5084 but not to *Pst* DC3000, as shown in e. Genomic DNA was extracted from the PL1‐ or GFP‐expressing leaves expressing 0 and 3‐day post‐infection, and the bacterial load was measured by qPCR relative to plant tissue. Error bars represent standard error of the mean of 3 independent replicates. Statistical significance within the same time points was calculated using a one‐way ANOVA and post hoc Tukey’s *T*‐test. Letters denote statistically significant groups (*P* < 0.05).

Previously, bacteriocins that are active against human pathogens have been expressed in *N. benthamiana* leaves and in leafy green vegetables (Schulz *et al.*, 2015; Paškevičius *et al.*, 2017; Schneider *et al.*, 2018). To express PL1 *in planta*, a construct that encodes PL1 with an N‐terminal 4 × c‐Myc tag was cloned into a Ti binary vector and transiently expressed in leaves of *N. benthamiana* using agroinfiltration. By 3‐days post‐infiltration, leaf extracts showed high levels of PL1 in western blots and high killing activity against PL1‐sensitive strains in spot tests. We estimated the quantities of PL1 within the infiltrated leaves by comparing in spot tests the killing activity of leaf extracts with PL1 standards produced in *E. coli*. When correlated with killing activity, PL1 levels *in planta* were equivalent to 0.35% of total plant protein (~5 µm), demonstrating that active PL1 can be produced efficiently in *N. benthamiana* leaves (Figure 1a,b).

After establishing that PL1 can be expressed transiently at high levels in leaves, we challenged these leaves with *Ps* to establish whether PL1 expression produced a qualitative difference in disease symptoms. Three days post‐agroinfiltration (now denoted as day 0), leaves were inoculated with *Ps* LMG5084 (a pathovar that is highly sensitive to PL1, see Table 1) or with *Pst* DC3000 (a PL1‐insensitive strain). Over the next 3 days, leaves were observed for symptom development and bacterial growth was measured. The infiltration of leaves with *Agrobacterium* has been shown to induce immune responses that inhibit the growth of *Ps* in subsequent inoculation (Zipfel *et al.*, 2006; Rico *et al.*, 2010; Love *et al.*, 2012). We therefore compared the growth of *Ps* in leaves that transiently express PL1 following agroinfiltration with that in leaves that transiently express green fluorescent protein (GFP), a non‐bactericidal protein. In PL1‐expressing leaves inoculated with *Ps* LMG5084, we observed a striking reduction in symptom severity (mild chlorosis only) compared with GFP‐expressing controls inoculated with *Ps* LMG5084, which exhibited black mottling and extensive necrosis by 7 days post‐infection (dpi) (Figure 1c).

When we measured bacterial load in *Ps* LMG5084‐inoculated leaves that express PL1*, Ps* titres were 5‐log units lower than in non‐agroinfiltrated control leaves; crucially, they were also 3‐log units lower than in leaves expressing GFP (Figure S3a). The process of syringe infiltration with buffer did not affect the growth of *Ps*; neither did GFP expression compared with the empty vector control (Figure S2). In PL1‐expressing leaves inoculated with the PL1‐resistant strain *Pst* DC3000, *Ps* titres were the same as in leaves expressing GFP (Figure S3b). However, titres of *Ps* LMG5084 (but not of *Pst* DC3000) that we recovered from leaves immediately following their inoculation (i.e. 0 dpi) were unexpectedly ~2‐log units lower in PL1‐expressing relative to GFP‐expressing leaves and non‐infiltrated controls (Figure S3a,b). Since all leaves were inoculated from a common bacterial suspension, the numbers of bacteria introduced into each leaf should have been the same for all samples. We suspected that the much lower titres recovered from PL1‐expressing leaves at 0 dpi (immediately after infiltration) were because bacteria were being killed post‐extraction as a result of the release of PL1 from cells during leaf grinding. We confirmed this by mixing leaf extracts from uninfected PL1‐expressing leaves with leaf extracts from infected non‐expressing leaves; here, we observed a reduction in *Ps* titres of ~2 log units with *Ps* LMG5084 (Figure S4a,b). To develop an alternative assay for bacterial titres *in planta,* we initially attempted to adapt the bioluminescence assay of Fan *et al. *(2008) to measure bacterial titres in leaves. However, in our hands *Ps* LMG5084, a field isolate, proved much more difficult to transform with the *lux*CDABE plasmid than did *Pst* DC3000. We therefore developed an alternative assay for bacterial load by using qPCR to measure the quantity of bacterial genomic DNA relative to plant DNA in leaf extracts (Ross and Somssich, 2016).

A standard curve showed a relationship between bacterial titres (colony forming units, CFU) and bacterial DNA recovered *in planta* that was near linear (Figure S5). This demonstrates that DNA levels provide a good proxy for measuring bacterial load. Therefore, we infected PL1‐ and GFP‐expressing leaves with *Ps* LMG5084 and *Pst* DC3000 and quantitated bacterial DNA using qPCR. By 3 dpi, we observed significantly reduced levels (*P* value = 0.031 by one‐way ANOVA) in PL1‐ compared with GFP‐expressing leaves following inoculation with *Ps* LMG5084 but not with *Pst* DC3000, consistent with the direct measure of bacterial titres (Figure 1d,e). Moreover, using DNA levels as a proxy for bacterial load does not distinguish between living and dead bacteria; therefore, this approach will inevitably overestimate the titres of living bacteria within the leaf and the genuine differences in bacterial load are likely to be even greater. In conclusion, we show that PL1 can be expressed to a high level in *N. benthamiana* leaves and that its expression correlates with both qualitative and quantitative disease resistance against the PL1‐sensitive strain *Ps* LMG5084 but not against the PL1‐insensitive strain, *Pst* DC3000.

### Expressing PL1 in Arabidopsis provides robust resistance against *P. syringae* pv. *syringae* LMG5084

**Figure 2:**
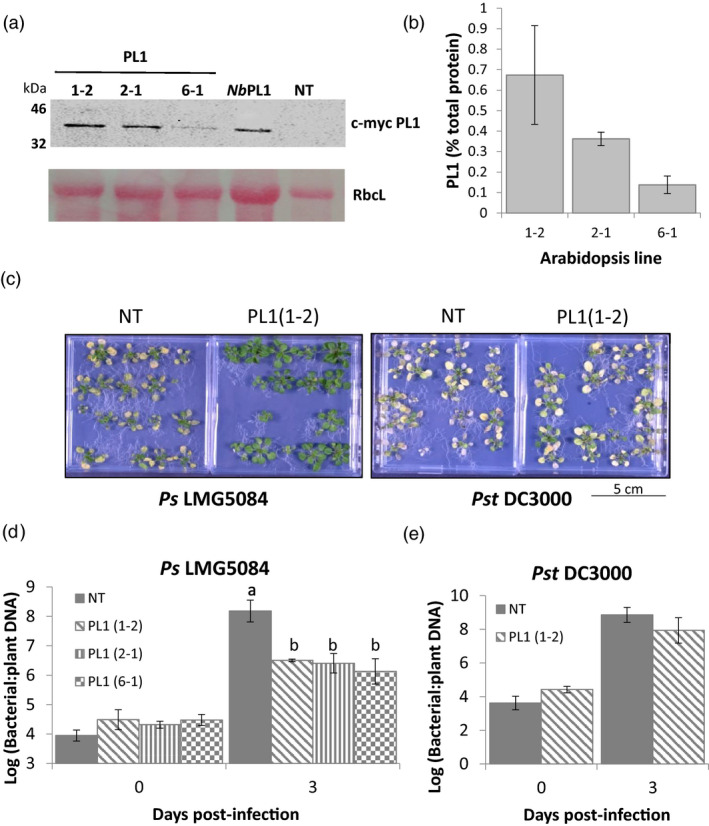
Growth of PL1‐susceptible but not of PL1‐resistant strains is attenuated in transgenic Arabidopsis plants that constitutively express PL1. a, PL1 can be expressed to high levels in Arabidopsis, as visualized by western blot analysis. PL1 levels in Arabidopsis whole seedlings obtained from 3 independent myc‐tagged PL1 transgenic lines (1‐2, 2‐1 and 6‐1). The corresponding Ponceau S staining of the large RuBisCO subunit (RbcL) was used as a loading control. A positive control (*Nb*PL1) from *N. benthamiana* and a negative control of a non‐transgenic Arabidopsis (NT) are also included on this blot. b, Serial dilutions of whole‐protein extracts obtained from each Arabidopsis PL1 transgenic line seedlings were spotted onto lawns of LMG5084 to estimate the percentage of PL1 activity. Error bars represent the standard deviation of 3 independent replicates. c, PL1‐expressing seedlings showed qualitative resistance to *Ps* LMG5084 but not to *Pst* DC3000. 14‐day‐old Arabidopsis PL‐expressing (PL1(1‐2), PL1(2‐1) and PL1(6‐1)), and non‐transgenic (NT) seedlings were flooded with either *Ps* LMG5084 or *Pst* DC3000 for 1 min, and symptoms were left to develop over 3 days. d, e, PL1‐expressing seedlings showed quantitative resistance to (d) *Ps* LMG5084 but not to (e) *Pst* DC3000. Leaves were collected 0 and 3‐day post‐infection, and genomic DNA was extracted from the leaves to estimate the bacterial load, as measured by qPCR, relative to plant tissue. Error bars represent the standard error of the mean of 4 independent replicates. Statistical significance within the same time points was calculated using a one‐way ANOVA and post hoc Tukey’s T‐test. Letters denote statistically significant groups (*P* < 0.05).

To better understand the efficacy of PL1‐mediated resistance, non‐transgenic (NT) Arabidopsis plants were transformed to express c‐myc PL1 and homozygous PL1‐expressing transgenic lines were selected. We characterized 3 independent lines, PL1(1‐2), PL1(2‐1) and PL1(6‐1), which exhibited levels of PL1 expression, varying from 0.13 to 0.67% of total protein (1.5−8.5 µm; Figure 2a,b). Over their lifespan, we observed no visible differences between any of the three transgenic lines and non‐transgenic (NT) plants with respect to size, general appearance, leaf morphology and flowering. PL1‐expressing transgenic lines were infected by spraying them with a suspension of the PL1‐susceptible pathovar, *Ps* LMG5084. As a control, the PL1‐resistant strain *Pst* DC3000 was used. Bacterial titres were then measured over 3 days. Titres of *Ps* LMG5084 were significantly lower in PL1(1‐2) and PL1(2‐1) (*P* < 0.001 and *P* < 0.004, respectively, by one‐way ANOVA) relative to NT controls. The greatest reduction in growth was observed in PL1(1‐2)_,_ the line with the highest levels of PL1 (Figure S6a). We observed no differences in the titres of *Pst* DC3000 between NT plants and any of the transgenic lines (Figure S6b).

As with *N. benthamiana*, although a common bacterial suspension was used to inoculate all plants, titres of bacteria recovered from PL1‐expressing lines immediately following inoculation with *Ps* LMG5084 were lower than expected on the basis of titre of the original inoculum (Figure S4). Again, we assume that live bacteria, recovered from plants at 0 dpi, were being killed by PL1 released from cells during grinding. We therefore measured bacterial growth by quantifying DNA levels by qPCR as previously used with *N. benthamiana* (Figure S7). Here, we carried out infections on 14‐day‐old seedlings grown on agar plates because disease phenotypes are more pronounced in younger plants (Zipfel *et al.*, 2004; Ishiga *et al.*, 2011). We observed striking differences in symptom severity between NT and all three PL1‐expressing transgenic lines. By 3 dpi, NT seedlings infected with either *Ps* LMG5084 or *Pst* DC3000 exhibited severe disease symptoms with most of the seedlings dead or dying (Figure 2c). In contrast, in all three PL1‐expressing lines, nearly all the *Ps* LMG5084‐infected seedlings appeared to be green and healthy (Figures 2c, S8), whereas *Pst* DC3000‐infected seedlings showed severe symptoms similar to those in NT plants (Figures 2c, S9). To quantify disease resistance, NT Arabidopsis and the 3 PL1‐expressing transgenic lines were infected by flooding plates with a suspension of bacteria and samples were taken at 0 and 3 dpi. At 3 dpi, the quantity of *Ps* LMG5084 DNA in the PL1‐expressing lines was ~1.5‐log units lower than in the NT control (Figure 2d); p values for PL1(1‐2), PL1(2‐1) and PL1(6‐1) were 0.002; 0.006; 0.006, respectively, showing that the differences are highly significant. Bacterial DNA levels in PL1(1‐2) seedlings infected with the PL1‐insensitive line *Pst* DC3000 were identical to levels in NT seedlings (Figure 2e). In conclusion, PL1 was able to provide strong qualitative and quantitative disease resistance to the PL1‐sensitive strain *Ps* LMG5084 but not to the PL1‐insensitive strain, *Pst* DC3000.

### PL1‐mediated resistance is not specific to *P. syringae* pv. *syringae* LMG5084

**Figure 3:**
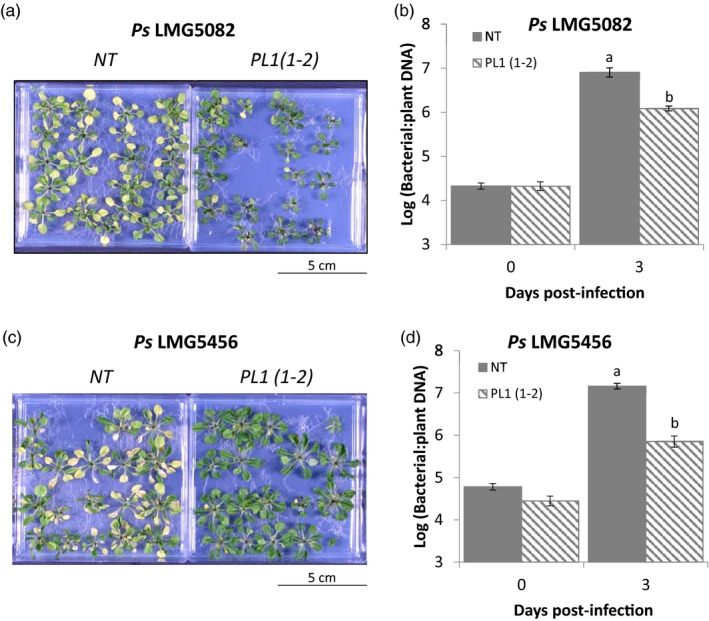
PL1‐mediated resistance is not specific to *Ps* LMG5084. Transgenic Arabidopsis lines show qualitative resistance to both PL1‐sensitive strains, *Ps* LMG5082 and *Ps* LMG5456. 14‐day‐old Arabidopsis non‐transgenic (NT) and PL 1‐expressing (PL1(1‐2)) seedlings were flooded with either a, *Ps* LMG5082 or c, *Ps* LMG5456 for 1 min. Symptoms were left to develop over 3 days. The PL1(1‐2) transgenic line showed quantitative resistance to both *Ps* LMG5082 and *Ps* LMG5456. To calculate bacterial DNA post‐infection, 14‐day‐old Arabidopsis NT and PL1(1‐2)) seedlings were flooded with either b, *Ps* LMG5082 or d, *Ps* LMG5456 for 1 min. Leaf samples were then collected 0 and 3 dpi. Bacterial DNA was extracted from them, and bacterial load was assessed by measuring the relative amount of bacterial DNA relative to plant DNA by qPCR. Error bars represent standard error of the mean of 4 independent replicates. Statistical significance within the same time points was calculated using a one‐way ANOVA and post hoc Tukey’s *T*‐test. Letters denote statistically significant groups (*P* < 0.05).

To demonstrate that the disease resistance mediated by PL1 expression *in planta* is not specific to a single *Ps* strain, we tested two additional PL1‐susceptible pathovars. We first established which of the remaining nine PL1‐sensitive strains could establish a compatible infection with Arabidopsis by flood‐infecting NT seedlings and screening them for characteristic *Ps* disease symptoms. From this test, we identified *Ps* pv. *syringae* LMG5082 and pv. *lachrymans* LMG5456 as suitable candidates. Both strains produced much less severe symptoms in PL1‐transgenic Arabidopsis compared with NT plants (Figure 3a,c; Figures S10 and S11). Also, relative to NT plants, bacterial DNA levels in transgenic lines were 0.8‐log units lower for *Ps* LMG5082 and 1.3‐log units lower for *Ps* LMG5456 (Figure 3b,d). Therefore, PL1‐mediated resistance is not confined to a single strain (*Ps* LMG5084) nor to *Ps*. pv. *syringae* pathovars.

### *P. syringae* mutants insensitive to PL1 lack LPS and show reduced virulence in PL1 transgenic Arabidopsis

**Figure 4:**
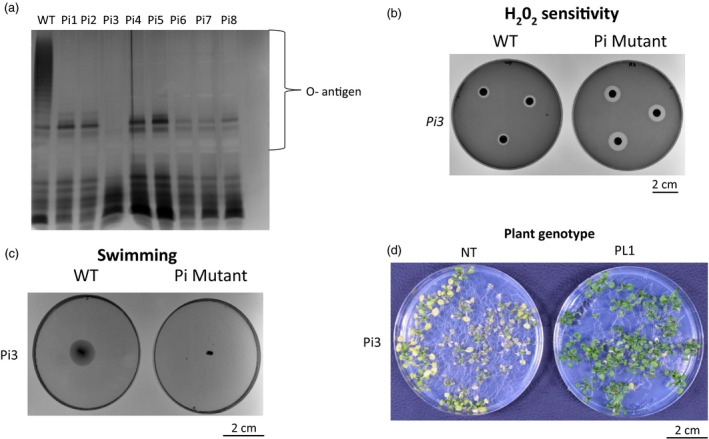
PL1‐tolerant *P. syringae* mutants have mutations in LPS biosynthesis genes and cannot overcome PL1‐mediated resistance in Arabidopsis. a, LPS extracted from PL1‐tolerant *Ps* lacks O‐antigen (PL1‐tolerant mutants are labelled Pi1 to Pi‐8). LPS of Pi1‐8 were visualised by SDS‐PAGE and silver staining. b, PL1‐tolerant *Ps* show increased sensitivity to hydrogen peroxide. Filter paper soaked with 1 % H_2_0_2_ was placed onto lawns of wild type (WT) and mutant *Ps* and incubated overnight. c, PL1‐tolerant *Ps* display defective motility. The swimming motility of WT and mutant *Ps* was assessed after 4 days of culture in 0.3% hrp‐derepressing media. d, PL1‐insensitive *Ps* can produce disease symptoms in non‐transgenic plants, but not in PL1‐transgenic plants. 14‐day‐old non‐transgenic (NT) or PL1 transgenic (PL1) seedlings were flooded with the mutant bacteria, and symptoms were observed after 3 days. The images shown are representative of the results obtained from three replicate experiments.

In our experiments, high levels of PL1 are produced *in planta*, which we predict will create a strong evolutionary pressure on *Ps* to acquire mutations conferring decreased sensitivity to PL1. Previous work has shown that LPS constitutes the primary receptor for the lectin‐like bacteriocins and that mutations in the LPS synthesis machinery can cause resistance to this class of protein antibiotics (Ghequire *et al.*, 2013, 2018; McCaughey *et al.*, 2014). To assess the robustness of protection against *Ps* that is provided by the *in planta* production of PL1, we first generated spontaneously arising PL1‐insensitive (Pi) mutants by growing *Ps* LMG5084 in liquid culture in rich media supplemented with 10 µm PL1. Surviving colonies were subcultured and eight independently arising *Ps* mutants that were highly tolerant to PL1 were selected for characterization. These mutants displayed only hazy zones of clearing at >10 µm PL1 in a spot test (Table S1). Whole‐genome sequencing was then performed to identify mutations that might be responsible for PL1 tolerance. All of the PL1‐tolerant lines carried mutations in genes that encode enzymes reported to be involved in LPS biosynthesis (Table S1). To investigate potential defects in LPS production, we purified and analysed LPS profiles isolated from *Ps* LMG5084 and from eight of the PL1‐tolerant mutant lines. Analysis by SDS‐PAGE and silver stain showed that each of these mutants lacks the outer membrane O‐antigen that is produced by the parental *Ps* LMG5084 strain (WT; Figure 4a). In addition, each of these mutants showed defects in motility, as measured in swimming assays and increased sensitivity to reactive oxygen species, as determined by exposure to 1% H_2_O_2_ (Figure 4b,c; Figures S12 and S13). Interestingly, when inoculated with the PL1‐tolerant mutants, NT Arabidopsis plants still developed severe symptoms similar to WT *Ps* LMG5084, but the transgenic PL1‐producing lines retained a healthy appearance suggesting that the latter retain effective resistance even to PL1‐tolerant *Ps* mutants (Figure 4d; Figure S14).

## Discussion

In this study, we set out to investigate whether the expression of bacteriocins *in planta* could be used as a strategy to confer resistance against bacterial infection. Here, we have established a proof‐of‐principle for bacteriocin‐mediated resistance against a key genus of plant pathogenic bacteria in two different model plant species. We are therefore optimistic that the concept of bacteriocin‐mediated crop protection is viable. Encouragingly, where bacteriocins have previously been assessed by the US FDA for safety as antibacterials for use in humans, they have been classified as ‘Generally Regarded as Safe’ (Schulz *et al.*, 2015). Furthermore, bacteriocins are narrow‐spectrum antimicrobial agents and they should therefore selectively target only specific plant pathogenic bacterial species and not affect the many commensal/mutually beneficial bacteria that persist in the plant rhizosphere; however, this requires further investigation (Mendes *et al.*, 2013). The combination of highly specific target range and negligible impact on benign species has been a crucial factor in the extensive worldwide adoption of BT‐insecticidal GM crops (Koch *et al.*, 2015) and we see parallels with the use of bacteriocins for protection against bacterial infections.

The use of novel peptides for defence against pathogens in agriculture is not a novel concept. Over 900 synthetic and natural AMPs have been characterized in the literature with a broad spectrum of effects including defence against pathogens, and there are several reports of transgene‐mediated overexpression being tested as strategy for conferring resistance to infection by plant pathogenic bacteria (De Souza Cândido *et al.*, 2014; Holaskova *et al.*, 2014; Ageitos *et al.*, 2017). For example, Hao *et al.* (2017) reported that expression of the AMP D2A21 conferred resistance against *Ps* pv. tabaci in transgenic tobacco, as evidenced by disease symptoms, although importantly the bacterial titres were not significantly reduced (Hao *et al.*, 2017). Transgenic expression of Bombinin (Zakharchenko *et al.*, 2018) and LfChimera (Chahardoli *et al.*, 2018) also conferred a degree of broad‐spectrum resistance against bacterial pathogens, but again, this was based primarily on symptom expression rather than a reduction in bacterial growth *in planta*. In contrast, the resistance conferred by expression of PL1 appears to be more robust and crucially more targeted against a narrow spectrum of specific bacterial pathovars. Moreover, the impact of AMPs on the root microbiome is still poorly understood; thus, the transgenic expression of biological agents like bacteriocins is advantageous because of their narrow target spectrum. Currently, there has been one study (involving wild tobacco plants expressing Mc‐AMP1 from *Mesembryanthemum crystallinum*) that suggested that expression of the peptide had a marginal effect on the root‐associated microbiota (Weinhold *et al*., 2018).

To future‐proof the use of bacteriocins in agriculture, the expression of heterologous cocktails of bacteriocin proteins would help to overcome the development of bacteriocin insensitivity and ensure the eradication of plant pathogenic bacteria (Wulff *et al.*, 2011). In this study, we show that lectin‐like bacteriocins represent promising candidates for transgenic expression. However, genome mining has identified additional classifications of bacteriocins in plant pathogenic bacteria, including tailocins, colicin‐M like bacteriocins and nuclease bacteriocins (Grinter *et al.*, 2012a). An important consideration when expressing bacteriocins *in planta* is the effect they might have on plant growth and development. Although we did not observe any obvious phenotypic effects of PL1‐expression, we will need to address this more thoroughly in the future. However, from our initial observations, we see no phenotypic differences between the transgenic and the NT plants used in this study. A key question for future studies to address is therefore whether the expression of PL1, or of other bacteriocins, negatively affects crop yield/quality.

A further consideration is the natural selection of bacterial populations that develop insensitivity to a bacteriocin; however, this can come with a fitness cost. For example, non‐pathogenic strains of Agrobacterium that express agrocin 84 can suppress the formation of crown gall by pathogenic strains in the field (Kerr and Htay, 1974; Ellis *et al.*, 1979). Our results suggest that for the PL1‐tolerant mutants tested here, which were isolated *in vitro*, the levels of PL1 exposure in the transgenic lines remained sufficient to offer robust resistance to infection, although perhaps surprisingly, these mutant lines retained virulence in NT plants. Possibly, the high bacterial titres in the flood inoculation method used in these experiments (much greater than titres that would normally be encountered in natural infections) are masking any fitness costs of a rough LPS phenotype such as the ability to survive out with the plant (epiphytic fitness) or infect in a biologically relevant manner. For example, mutations resulting in the loss of o‐antigen in *Ps* pv. *syringae* 61 cause a reduction in virulence in bean pods when inoculated with a toothpick (Deng *et al.*, 2010). Moreover, although LPS mutations associated with PL1 tolerance should diminish the ability of PL1 to dock to the bacterial cells, they will not affect the interaction with BamA which is the primary biological target. This could explain why the transgenic plants retain resistance to the PL1 tolerant mutants, particularly given the high levels of bacteriocin present.

Bacteriocin production is not exclusive to the genus *Pseudomonas* and so this strategy should in principle be applicable to a wide variety of important phytopathogens, such as *Xanthomonas *spp. (which causes the greening and blight of rice and banana; Pham *et al.*, 2004; Ghequire *et al.*, 2012); *Ralstonia solanacearum* (which causes potato brown rot and bacterial wilt of tomato; Huerta *et al.*, 2015) and *Pectobacterium and Dickeya *spp. (which cause potato soft rot and blackleg; Roh *et al.*, 2010; Chan *et al.*, 2011; Grinter *et al.*, 2012b; Czajkowski *et al.*, 2013)*.*

We propose that bacteriocin‐mediated resistance in plants represents a technology that can be utilized to control bacterial pathogens in agronomically important crops. Critically, plant‐bacterial ecosystems are dynamic and complex, therefore, we expect that their great genomic diversity will promote bacteriocin evolution and hence provide a very large, exploitable resource for future applications.

## Experimental procedures

### Bacterial strains

*Ps* isolates (Table 1) were obtained from the National Collection of Plant Pathogenic Bacteria and the Belgian Coordinated Collections of Microorganisms (LMG). *Ps* strains were cultured at 28°C in Kings broth B (KB) media, 20 g/L peptone, 1.5 g/L K_2_PO_4_, 1.5 g/L MgSO_4_ and 10 mL/L glycerol (pH 7.5).

### Motility experiments

For the motility swimming assay, an overnight liquid culture of *Ps* was stabbed into 0.3 % hrp‐derepressing minimal media (10 mm sucrose, 50 mm potassium phosphate buffer, 7.6 mm (NH_4_)_2_SO_4_, 1.7 mm MgCl_2_, 1.7 mm NaCl, pH 5.7) agar and incubated at 28°C for 4 days (Huynh *et al.*, 1989; Deng *et al.*, 2010).

### LPS extraction

A 2 mL bacterial culture with an OD_600_ = 1 was pelleted by centrifugation and washed in 10 mm MgCl_2_ (to remove any trailing media). LPS was then extracted using an LPS extraction kit (iNtRON Biotechnology, Gyeonggi, South Korea). The LPS pellet was resuspended in 50 µL of 10 mm Tris, pH 8. To ensure complete solubilization of the LPS pellet, the sample was boiled at 95 °C for 2 min and was further treated with 3 µg/µL of proteinase K at 50 °C for 30 min to obtain highly pure LPS from bacterial cells.

### Plant growth conditions

*Nicotiana benthamiana* plants were grown using long‐day conditions, consisting of 16 h light/8 h dark (at 26 and 18 °C, respectively) at 60% humidity and at a light level of 80 μmol/m^2^/s. Arabidopsis plants were grown in short‐day conditions at light level of 80 µmol/m^2^/s, consisting of 9 h light/15 h dark (at 22 and 18 °C, respectively) at 60/70% humidity.

### Gene cloning

To express PL1 in *E. coli*, the PL1‐encoding sequence (with no stop codon) was amplified using standard PCR reactions, a high fidelity Phusion Taq polymerase enzyme (New England Biolabs, Hitchin, UK) and appropriate templates, followed by cloning into *NdeI‐XhoI* sites in the pET21 vector (McCaughey *et al.*, 2014). For constitutive transgene‐mediated expression *in planta,* the PL1 coding sequence was fused to an N‐terminal 4xMyc tag and cloned into the *KpnI* site of pJO530, a derivative of pBIN19 (Cecchini *et al.*, 1997). A Ti plasmid vector that expresses GFP (Haseloff *et al.*, 1997) was used as a control for transient assays in *N. benthamiana* (Cecchini *et al.*, 1997). These plasmids are denoted pJOPL1 and p35S‐GFP, respectively. Plasmids used in this study were linearized by digestion using the appropriate restriction enzymes (New England Biolabs, Hitchin, UK). All DNA constructs were verified by sequencing (Source Bioscience, Oxford, UK).

### Expression and purification of PL1

Expression and purification were carried out according to McCaughey *et al.* (2014). Briefly, the pET21 plasmid containing PL1 was transformed into BL21 DE3 pLyS cells (Agilent, Edinburgh, UK). PL1 expression was induced at mid‐log phase by supplementing the media with 0.3 mm isopropyl β‐D‐1‐thiogalactopyranoside (IPTG), and the cells were grown at 22 °C for 20 h and harvested by centrifugation. The cells were lysed using an MSE Soniprep 150 (Wolf Laboratories, York, UK) and the cell‐free lysate was applied to a 5 mL HisTrap HP column (GE Healthcare, Amersham, UK), and PL1 was eluted using a 5–500 mm imidazole gradient. The remaining contaminants were removed by gel filtration chromatography on a Superdex S75 26/600 column (GE Healthcare, Amersham, UK). PL1 was concentrated using a centrifugal concentrator (Vivaspin 20, Epsom, UK) with a 5 kDa molecular weight cut‐off and stored at −80 °C.

### Soft agar overlay susceptibility assays

Soft agar overlay spot assays were performed using the method of Fyfe *et al.* (1984). Fifty microlitres of test strain culture at mid‐log was inoculated in 0.8 % soft agar and poured over a KB agar plate, as appropriate. 5 µL of undiluted and serially diluted bacteriocin solution/plant protein extract was spotted onto the plates and incubated for 20 h at 28 °C, after which time the plates were inspected for zones of bacterial growth inhibition.

### Transgene expression in *planta*

Transgene expression of the T‐DNA constructs *in planta* was achieved by transforming the constructs into *Agrobacterium tumefaciens* (strain GV3101). *N. benthamiana* leaves were infiltrated with GV3101 containing the appropriate vector (Kapila *et al.*, 1997). For Arabidopsis transformations, plants were floral dipped according to Zhang *et al.* (2006)*.* PL1 expression was detected using western blots. Protein was extracted by macerating frozen leaf tissue in 20 mm Tris‐HCl, 200 mm NaCl, pH 7.5, supplemented with cOmplete™, EDTA‐free Protease Inhibitor Cocktail (Roche, West Sussex, UK), and the protein concentration of the supernatant was determined by Bradford assay (BioRad, Perth, UK). Ten micrograms of protein extract was separated on 16% SDS‐PAGE and transferred onto a PVDF membrane. Proteins were detected using an anti‐c‐myc monoclonal antibody (sc‐40; Santa Cruz Biotech, Texas) and an anti‐mouse HRP conjugate antibody (W4021; Promega, Southampton, UK).

### Infection assays *in planta*

For infection studies in *N. benthamiana*, 1 × 10^5^ CFU/mL of *Ps* was syringe‐infiltrated into selected leaves of 4‐6‐week‐old plants, according to Hann and Rathjen (2007). For Arabidopsis, a suspension of 1 × 10^8^ CFU/mL of *Ps* supplemented with 0.025% Silwet L‐77 (Lehle Seeds, Texas) was sprayed onto the leaves of 6‐week‐old plants until they were visibly wet (Ishiga *et al.*, 2011). For the flood inoculation method, agar plates containing 14‐day‐old Arabidopsis seedlings were flooded for 1 min with a bacterial suspension comprising 1 × 10^6^ CFU/mL of bacteria supplemented with 0.025% Silwet L‐77 (Lehle Seeds, Texas). Plates containing transgenic seedlings were supplemented with 15 µg/mL of hygromycin B (Sigma‐Aldrich, Gillingham, UK).

### Bacterial titre assay using qPCR

DNA was extracted from infected *N. benthamiana* leaves using a DNAzol kit (Thermo Fisher Scientific, Paisley, UK) according to the manufacturer’s protocol. To extract DNA from infected Arabidopsis seedlings, plant tissue was frozen in liquid nitrogen and the DNA was extracted using FastDNA™ SPIN Kit for Soil (MP Biomedicals, California). Bacterial and plant DNA levels were quantified using qPCR, essentially as described by Love *et al.* (2007). qPCR was performed in an Applied Biosystems StepOnePlus Real‐Time PCR System (Thermo Fisher Scientific, Paisley, UK), using Fast SYBR™ Master Mix (Thermo Fisher Scientific, Paisley, UK) and 0.16 μm of primers. Bacterial DNA levels *in planta* were determined using primers specific for the *Ps oprF* gene (Ross and Somssich, 2016) and were normalized against the *ACT2* (AT3G18780) gene in Arabidopsis or the 18S rRNA genes in *N. benthamiana* (Love *et al.*, 2007)*.* A list of the PCR primers used in this study is provided in Table S2.

### Isolation and whole‐genome sequencing and analysis of PL1‐insensitive *Ps* strains

Fifty microlitre aliquiots of a *Ps* LMG5084 overnight culture were pelleted at 3000 ***g*** for 10 min and re‐suspended in 1 mL of 10 µm PL1. The bacteria were then incubated at 28 °C for 4 h, plated out on KB plates, and incubated overnight. DNA was extracted from wild type *Ps* LMG5084 and its PL1‐insensitive mutants using the GenElute Bacterial Genomic DNA Kit (Sigma‐Aldrich). Libraries were prepared with the NEBNext Ultra II library kit, according to the manufacturer’s instructions, and sequenced on the Illumina HiSeq × platform to obtain 150 bp paired‐end reads with an average depth of 40‐fold. Raw sequences from this study have been deposited in the European Nucleotide Archive (ENA) under the accession numbers detailed in Table S3. *Ps* LMG5084 wild type was assembled using Velvet v 1.2 with multiple assemblies generated using VelvetOptimser v 2.2.5 (Zerbino and Birney, 2008; Zerbino, 2010; Page *et al.*, 2016). The assembly with the best N_50_ was subjected to assembly improvement. Contigs were ordered using Mauve v 2.4.0, scaffolded with SPPACE v 2.0‐1 and gaps filled with GapFiller v 1.11‐1 (Rissman *et al.*, 2009; Boetzer *et al.*, 2011; Boetzer and Pirovano, 2012). The assembly was then annotated using Prokka v 1.5 and Roary v 3.11.2 based on the reference *Ps* B728a accession no. NC_007005.1 (Seemann, 2014; Page *et al.*, 2015). This reference genome was chosen according to the top species hit from Kraken (Wood and Salzberg, 2014). For *Ps* LMG5084 PL1‐insensitive mutants, sequence reads were mapped to *Ps* LMG5084 wild type using BWA v 0.7.17. SNPs were called and filtered using SAMtools mpileup and BCFtools v 0.1.19 (Li, 2011). Variant calls were then filtered and retained if the depth was greater than 5, quality greater than 50, mapping quality greater than 20, and the depth of reference (forward and reverse) reads was greater than the depth of alternative reads. Variant effects were predicted using SnpEff v 4.3T (Cingolani *et al.*, 2012).

### Statistical analysis

Statistical analysis used in this study was performed with Minitab 17 statistical software using one‐way ANOVA followed by Tukey’s multiple comparison test or a Dunnett’s test.

## Conflicts of Interest

A PCT patent (PCT/EP2018/057826) was submitted on behalf of the University of Glasgow in March 2018 to the European patent office.

## Author Contributions

WMR and RWG carried out the experimental work and contributed to the writing of the manuscript. AC and JP contributed to the genomic sequencing of *P. syringae* pathovars and mutants. DCW and JJM directed the project and contributed to the writing of the manuscript.

## Accession Numbers

**Table:** 

Sanger ID	Sample	ENA accession number
4526STDY7070045	*Ps* LMG 5084 Wild type	SAMEA104233059
4526STDY7070060	*Ps* LMG 5084 Pi1	SAMEA104233065
4526STDY7070076	Ps LMG 5084 Pi2	SAMEA104233071
4526STDY7070084	*Ps* LMG 5084 Pi3	SAMEA104233074
4526STDY7070092	*Ps* LMG 5084 Pi4	SAMEA104233077
4526STDY7070100	*Ps* LMG 5084 Pi5	SAMEA104233080
4526STDY7070108	*Ps* LMG 5084 Pi6	SAMEA104233083
4526STDY7070116	*Ps* LMG 5084 Pi7	SAMEA104233086
4526STDY7070037	*Ps* LMG 5084 Pi8	SAMEA104233055

ENA accession numbers for sequenced samples

## Supporting information


**Figure S1** PL1 activity on a spot test against a PL1‐sensitive strain of *Ps*
**Figure S2** Pre‐infiltration with Agrobacterium primes plant immunity when challenged with *Ps*.
**Figure S3** PL1 expression in *N. benthamiana* attenuates growth of LMG5084 but not DC3000 as (as *determined* by cfu counting).
**Figure S4** PL1 *expression* *in planta* affects bacterial recovery as determined by cfu counting.
**Figure S5** *Bacterial* titres in *N. benthamiana* leaves correlate with recovery of bacteria DNA from plant tissue.
**Figure S6** Arabidopsis‐expressing PL1 attenuates the growth of *Ps* LMG5084 but not *Pst* DC3000 (measured by cfu counting).
**Figure S7** Bacterial titres in Arabidopsis tissue correlate with recovery of bacteria DNA from plant tissue.
**Figure S8** PL1 expression in Arabidopsis seedlings provides robust disease resistance against *Ps* LMG5084.
**Figure S9** PL1 expression in Arabidopsis seedlings does not provide robust disease resistance against *Pst* DC3000.
**Figure S10** PL1 *expression* provides robust disease resistance against *Ps* LMG5082.
**Figure S11** PL1 *expression* provides robust disease resistance against *Ps* LMG5456.
**Figure S12** PL1‐*insensitive* strains are deficient in swimming compared with the wild type.
**Figure S13** PL1‐*insensitive* strains are more sensitive to 1% hydrogen peroxide.
**Figure S14** PL1‐insensitive strains of *Ps* LMG5084 cannot induce disease symptoms in transgenic plants expressing PL1.
**Table S1** *Mutations* linked with PL1‐resistance.
**Table S2** qPCR *primers* used in this study.
**Table S3** ENA *accession* numbers for sequenced samples.
