# Supplementary material for: Engineering bacteriocin‐mediated resistance against the plant pathogen Pseudomonas syringae
Source: Plant Biotechnol J. 2019 Dec 3;18(5):1296–306. doi: 10.1111/pbi.13294 (PMC7152609; doi:10.1111/pbi.13294)
Supplement: Supplementary file 1 — Figure S1 PL1 activity on a spot test against a PL1‐sensitive strain of Ps Figure S2 Pre‐infiltration with Agrobacterium primes plant immunity when challenged with Ps. Figure S3 PL1 expression in N. benthamiana attenuates growth of LMG5084 but not DC3000 as (as determined by cfu counting). Figure S4 PL1 expression in planta affects bacterial recovery as determined by cfu counting. Figure S5 Bacterial titres in N. benthamiana leaves correlate with recovery of bacteria DNA from plant tissue. Figure S6 Arabidopsis‐expressing PL1 attenuates the growth of Ps LMG5084 but not Pst DC3000 (measured by cfu counting). Figure S7 Bacterial titres in Arabidopsis tissue correlate with recovery of bacteria DNA from plant tissue. Figure S8 PL1 expression in Arabidopsis seedlings provides robust disease resistance against Ps LMG5084. Figure S9 PL1 expression in Arabidopsis seedlings does not provide robust disease resistance against Pst DC3000. Figure S10 PL1 expression provides robust disease resistance against Ps LMG5082. Figure S11 PL1 expression provides robust disease resistance against Ps LMG5456. Figure S12 PL1‐insensitive strains are deficient in swimming compared with the wild type. Figure S13 PL1‐insensitive strains are more sensitive to 1% hydrogen peroxide. Figure S14 PL1‐insensitive strains of Ps LMG5084 cannot induce disease symptoms in transgenic plants expressing PL1. Table S1 Mutations linked with PL1‐resistance. Table S2 qPCR primers used in this study. Table S3 ENA accession numbers for sequenced samples. [file PBI-18-1296-s001.docx]

**Supplementary Figures and Tables**


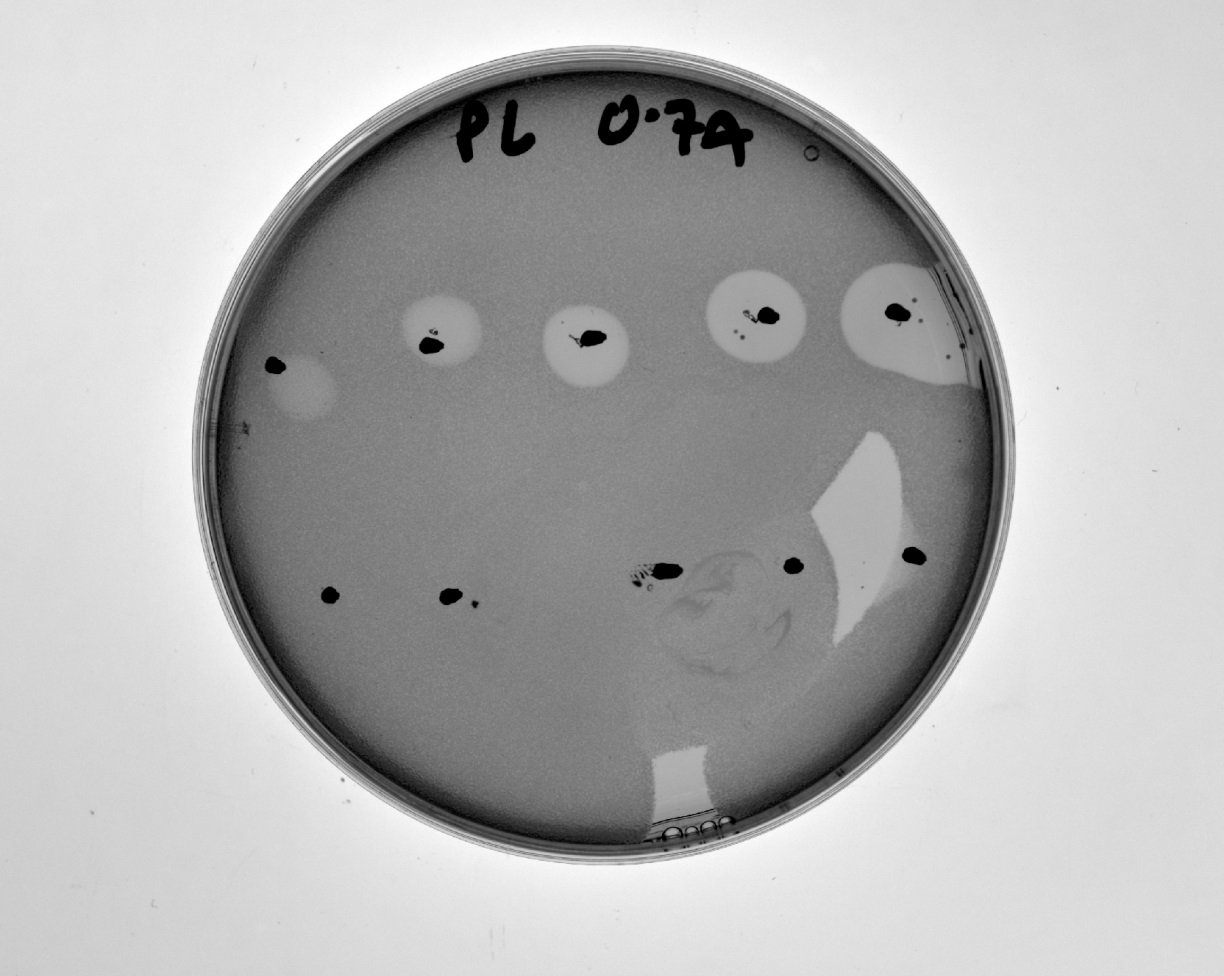


19 3.8 0.76 0.152 0.0304 µM

**Figure S1. PL1 activity on a spot test against a PL1-sensitive strain of *Ps*.** Serial dilutions of PL1 were spotted onto lawns of the PL1-sensitive strain *Ps* LMG5084.


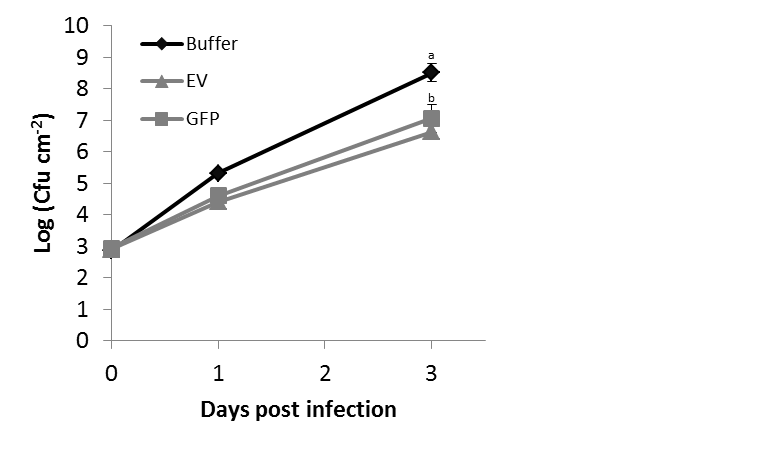


**Figure S2. Pre-infiltration with Agrobacterium primes plant immunity when challenged with *Ps*.** *N. benthamiana* leaves were agroinfiltrated with either buffer (diamonds), *Agrobacterium* containing an empty vector or a vector expressing GFP. Plants were then infected with *Ps* LMG5084 3 days post infiltration, leaf samples were taken 0, 1 and 3 dpi and the bacterial load (cfu cm^-2^) was measured. Error bars represent standard error of the mean of 3 independent replicates. Statistical significance within the same time points was revealed using a 1-way ANOVA post hoc Tukey T-test. Letters denote statically significant groups (p< 0.05).


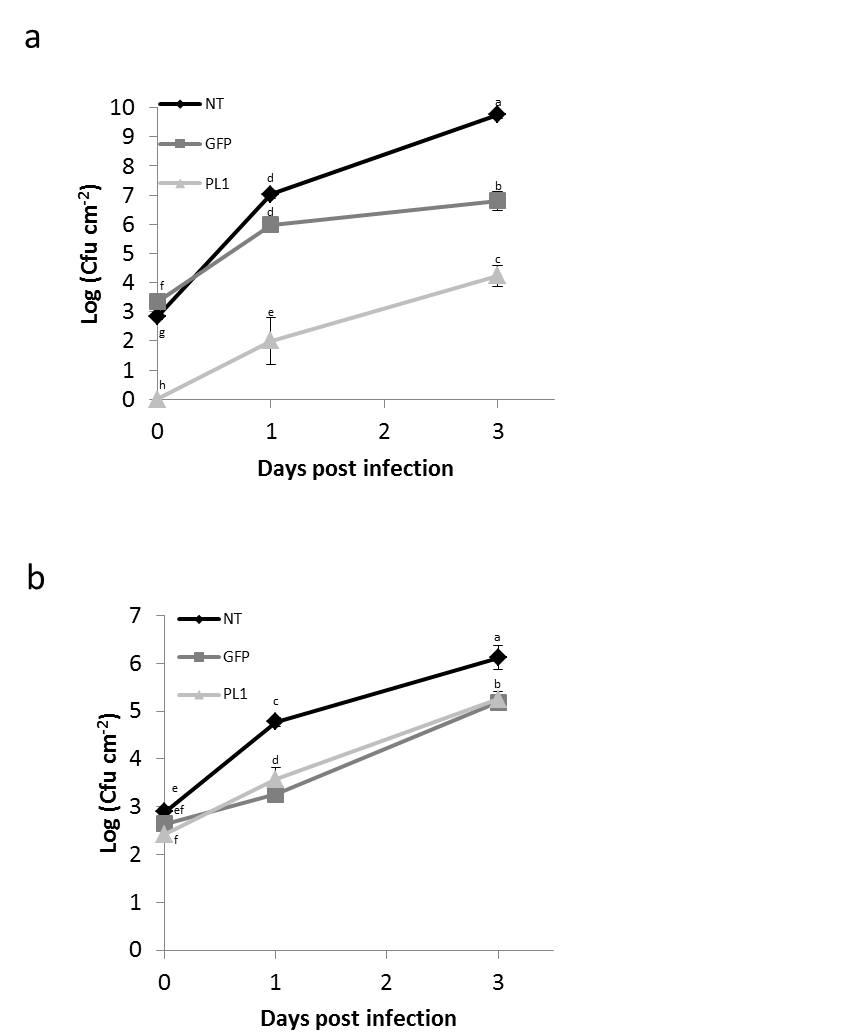


**Figure S3. PL1 expression in *N. benthamiana* attenuates growth of LMG5084 but not DC3000 as (as determined by cfu counting).** *N. benthamiana* leaves expressing PL1, GFP or non-agroinfiltrated controls were infected with **a,** *Ps* LMG5084 or **b,** DC3000**.** Leaf samples were taken 0, 1 and 3 days post infection and the bacterial load (cfu cm^-2^) was measured. Error bars represent standard error of the mean of 3 independent replicates. Statistical significance within the same time points was revealed using a 1-way ANOVA post hoc Tukey T-test. Letters denote statically significant groups (p< 0.05).


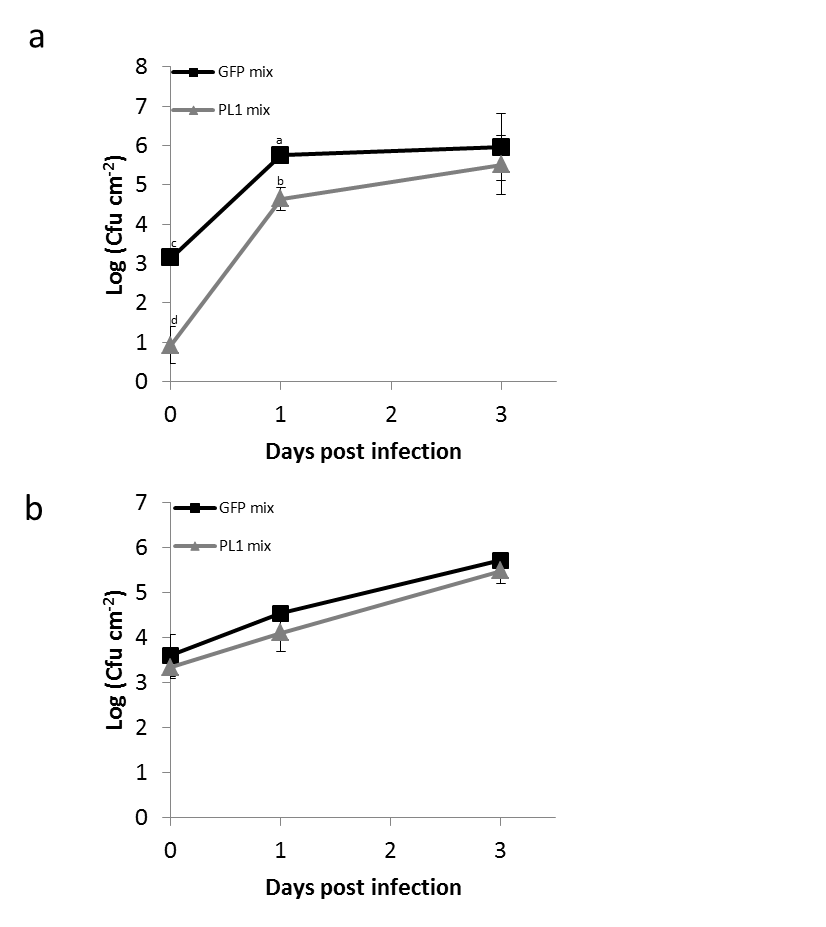


**Figure S4. PL1 expression *in planta* affects bacterial recovery as determined by cfu counting.** *N. benthamiana* leaves transiently expressing GFP were syringe-infiltrated with **a,** *Ps* LMG5084 or **b,** DC3000. Leaf discs were taken 0, 1 and 3 days post infection and mixed with a leaf disc of either a PL1-expressing or an un-infiltrated leaf and, the bacterial loads were (cfu cm-^2^) measured. Error bars represent standard error of the mean of 3 independent replicates. Statistical significance within the same time points was revealed using a 1-way ANOVA post hoc Tukey T-test. Letters denote statically significant groups (p< 0.05).


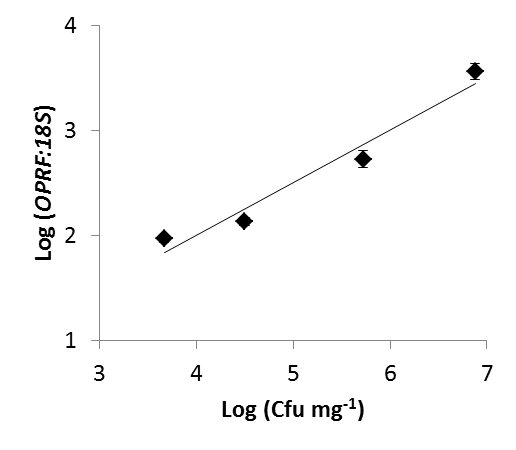


**Figure S5. Bacterial titres in *N. benthamiana* leaves correlates with recovery of bacteria DNA from plant tissue.** Serial dilutions of bacteria were infiltrated into *N. benthamiana* leaves and bacterial DNA was immediately extracted from plant tissue. Levels of bacterial *DNA* relative to *N. benthamiana* DNA were measured using qPCR. Error bars represent standard error of the mean of 3 independent replicates.


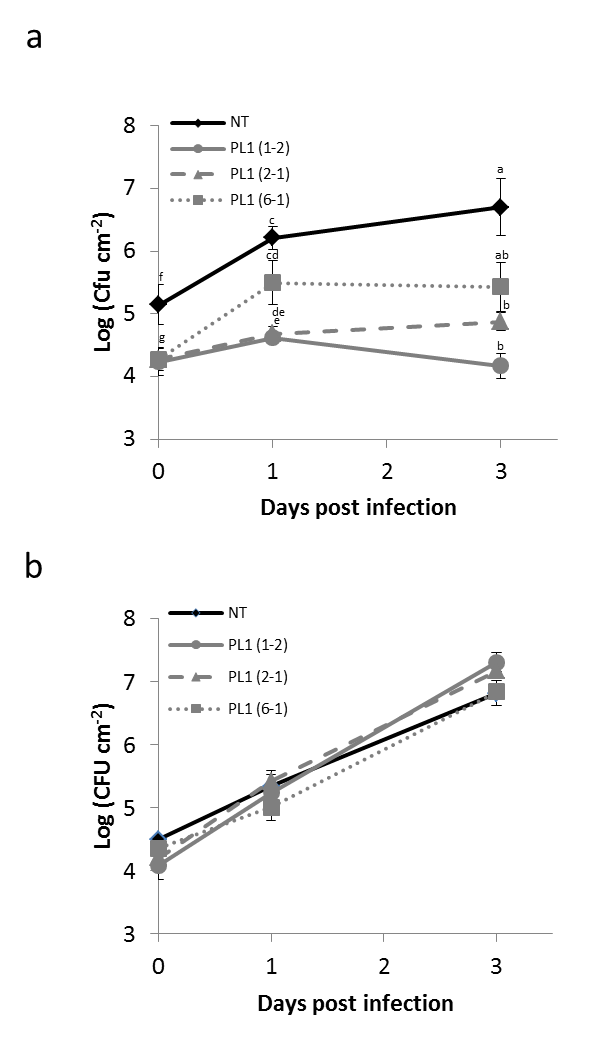


**Figure S6. Arabidopsis expressing PL1 attenuates the growth of LMG5084 but not DC3000 (measured by cfu counting).** Three independent PL1 expressing lines, PL1(1-2), PL1(2-1) and PL1(6-1) and a NT control were spray inoculated with 1 x 10^8^ CFU mL^-1^ of either **a,** *Ps* LMG5084 or **b,** DC3000. Leaf samples were taken 0, 1 and 3 days post infection to measure the bacterial load (cfu cm^-2^). Error bars represent standard error of the mean of 3 independent replicates. Statistical significance within the same time points was revealed using a 1-way ANOVA post hoc Tukey T-test. Letters denote statically significant groups (p< 0.05).


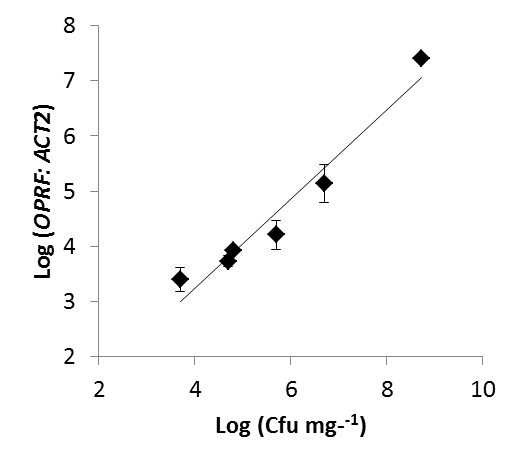


**Figure S7. Bacterial titres in Arabidopsis tissue correlates with recovery of bacteria DNA from plant tissue.** Serial dilutions of bacteria were infiltrated into leaves and bacterial DNA was immediately extracted from plant tissue. Levels of bacterial DNA relative to Arabidopsis DNA were measured using qPCR. DNA. Error bars represent standard error of the mean of 3 independent replicates.


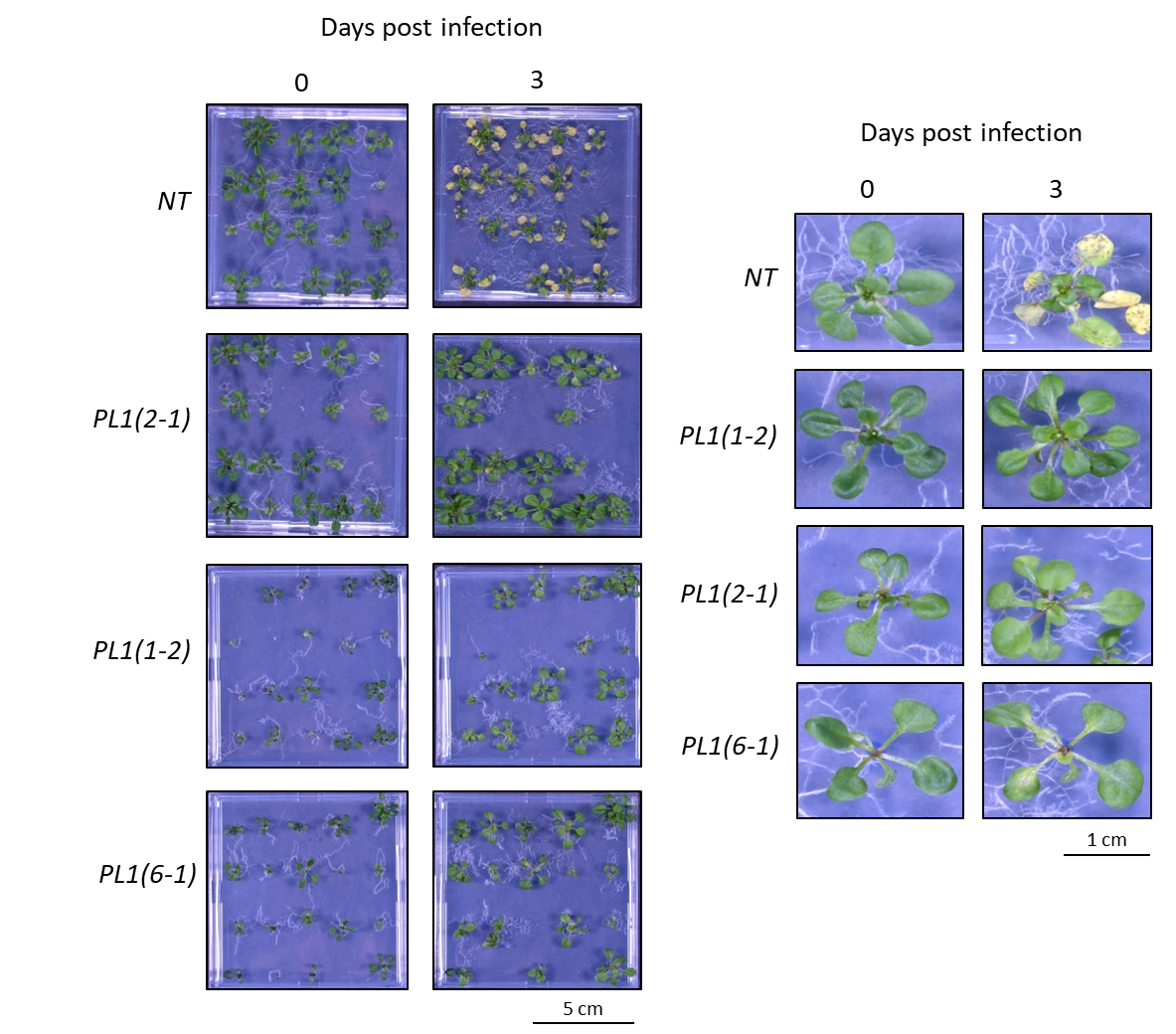


**Figure S8. PL1 expression in Arabidopsis seedlings provides robust disease resistance against LMG5084.** Fourteen-day-old non-transgenic (NT) and 3 independent PL1 transgenic seedlings were flood inoculated with 1 x 10^6^ CFU mL^-1^ of *Ps* LMG5084 and symptoms were left to develop other 3 days. Pictures of plates and individual plants were taken 0- and 3-days post infection. Experiments were repeated 3 times with identical results.


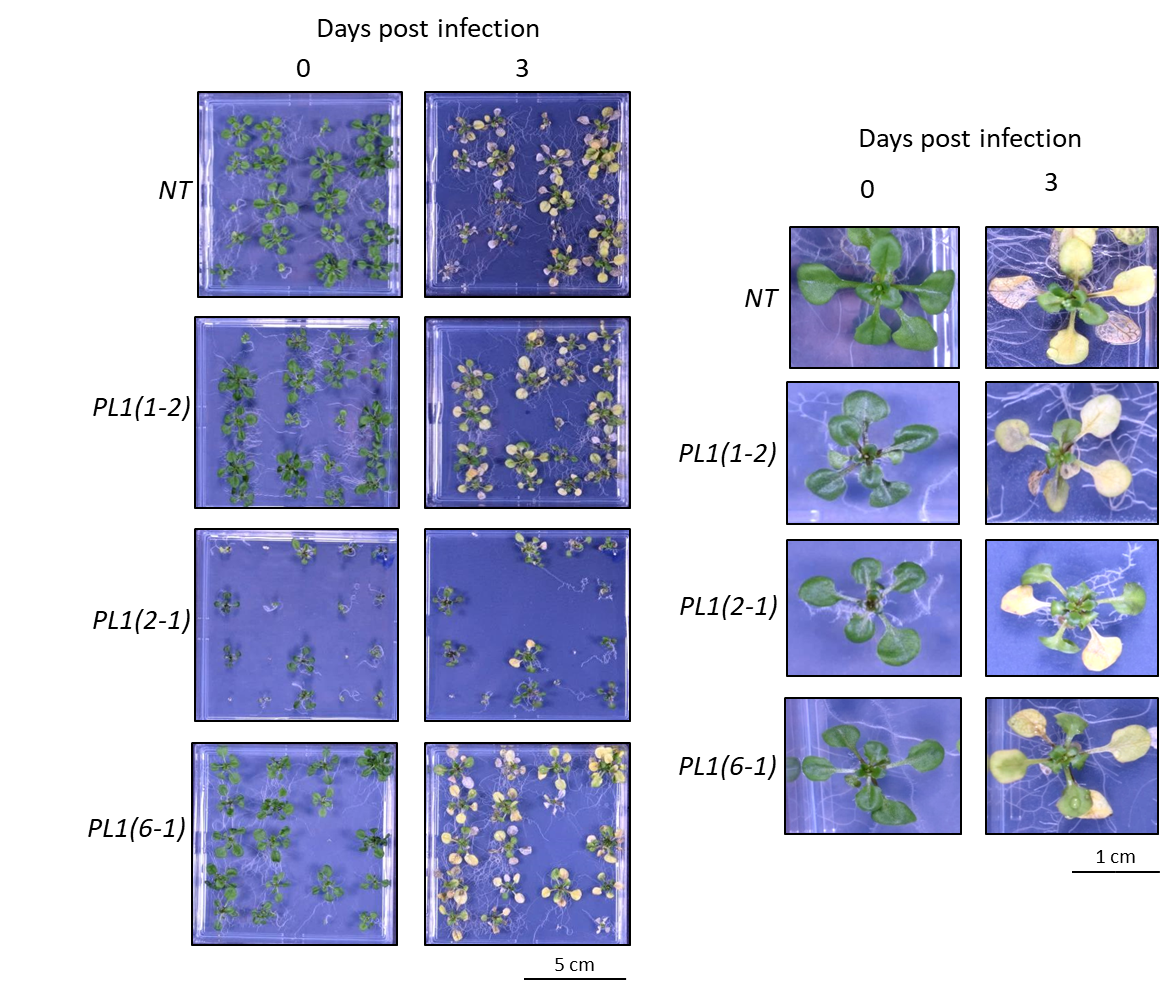


**Figure S9. PL1 expression in Arabidopsis seedlings does not provide robust disease resistance against DC3000.** Fourteen-day-old non-transgenic (NT) and 3 independent PL1 transgenic seedlings were flood inoculated with 1 x 10^6^ CFU mL^-1^ of *Ps* DC3000 and symptoms were left to develop other 3 days. Pictures of plates and individual plants were taken 0- and 3-days post infection. Experiments were repeated 3 times with identical results.


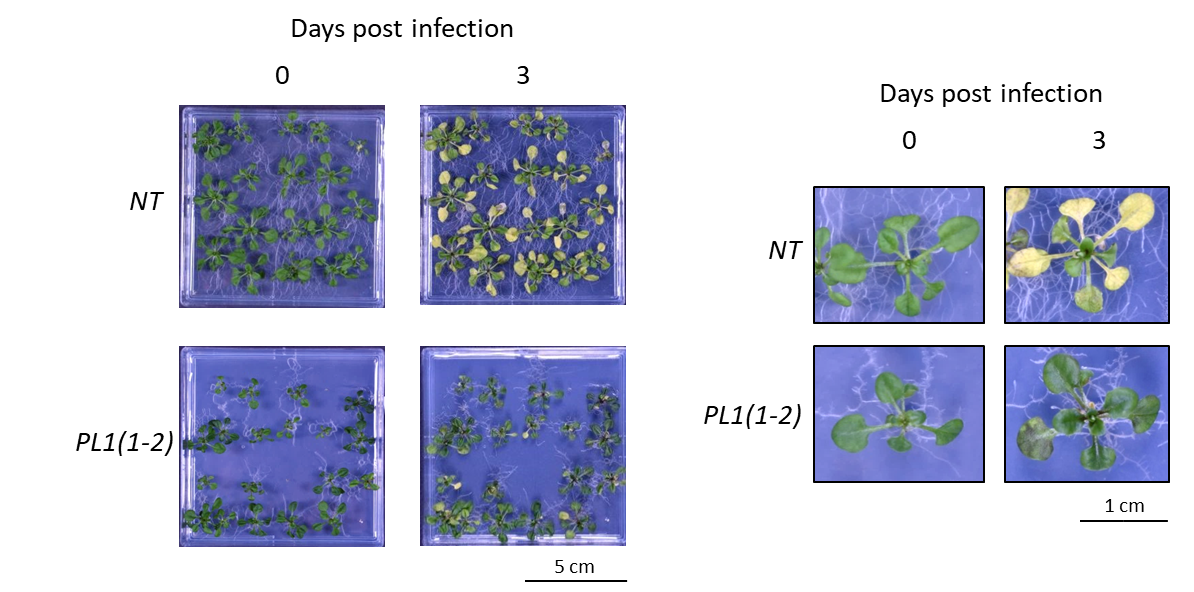


**Figure S10. PL1 expression provides robust disease resistance against LMG5082.** Fourteen-day-old non-transgenic (NT) and transgenic seedlings (PL1(1-2)) were flood inoculated with 1 x 10^6^ CFU mL^-1^ of *Ps* LMG5082 and symptoms were left to develop other 3 days. Pictures of plates and individual plants were taken 0- and 3-days post infection. Experiments were repeated 3 times with identical results.


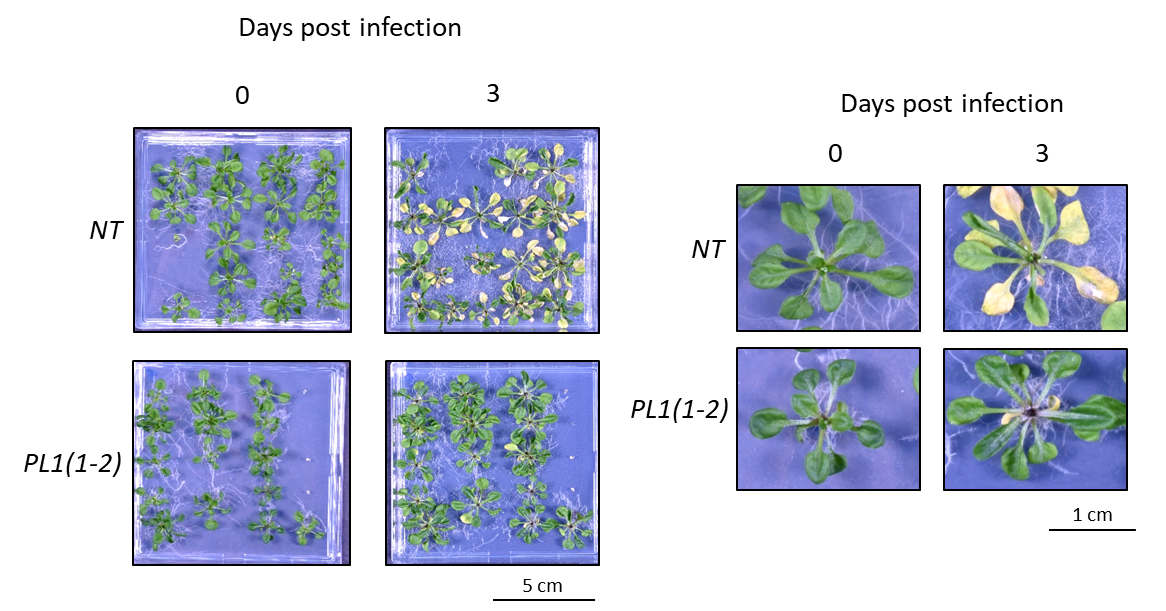


**Figure S11. PL1 expression provides robust disease resistance against LMG5456.** Fourteen-day-old non-transgenic (NT) and transgenic seedlings (PL1(1-2)) were flood inoculated with 1 x 10^6^ CFU mL^-1^ of *Ps* LMG5456 and symptoms were left to develop other 3 days. Pictures of plates and individual plants were taken 0- and 3-days post infection. Experiments were repeated 3 times with identical results.


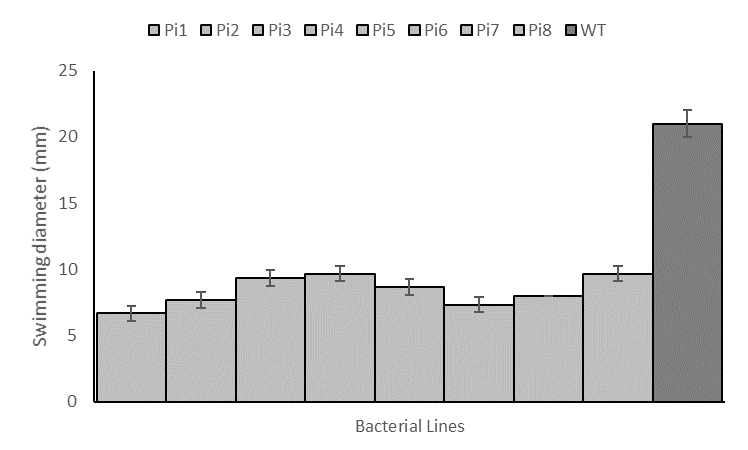


a

a

a

a

a

a

a

a

**Figure S12. PL1-insensitive strains are deficient in swimming compared to the wild-type.**

Bacterial cultures were inoculated into HRP de-repressing media supplemented with 0.3% agar and incubated at 24 °C for six days. Swimming was assessed by measuring average colony diameter. Error bars represent the standard deviation of 3 independent experiments. Statistical significance for the PL1 insensitive mutants compared to the WT was assessed by a 1 way-ANOVA, post hoc- Dunnet’s T-test. Swimming diameter which is statistically different (p<0.05) from the WT is denoted with a letter.


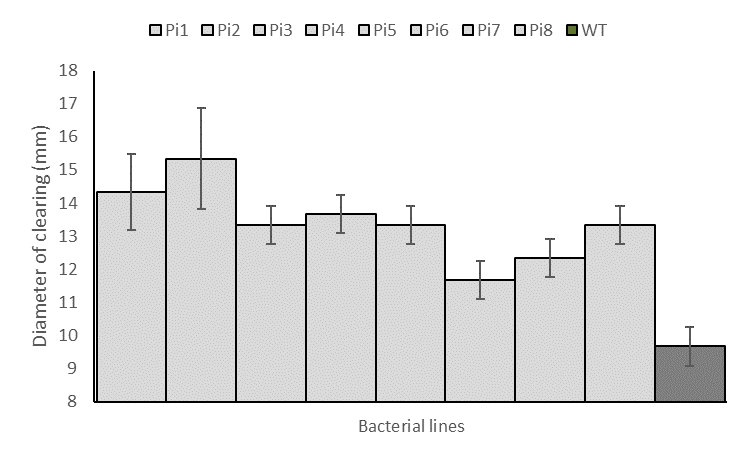


a

a

a

a

a

a

a

a

**Figure S13. PL1-insensitive strains are more sensitive to 1% hydrogen peroxide.** Whatman paper socked in 1% H_2_0_2_ were placed on lawns of bacteria and incubated at 24 °C overnight. Error bars represent the standard deviation of 3 independent experiments. Statistical significance for the PL1 insensitive mutants compared to the WT was assessed by a 1 way-ANOVA, post hoc- Dunnet’s T-test. Swimming diameter which is statistically different (P < 0.05) from the WT is denoted with a letter.

**
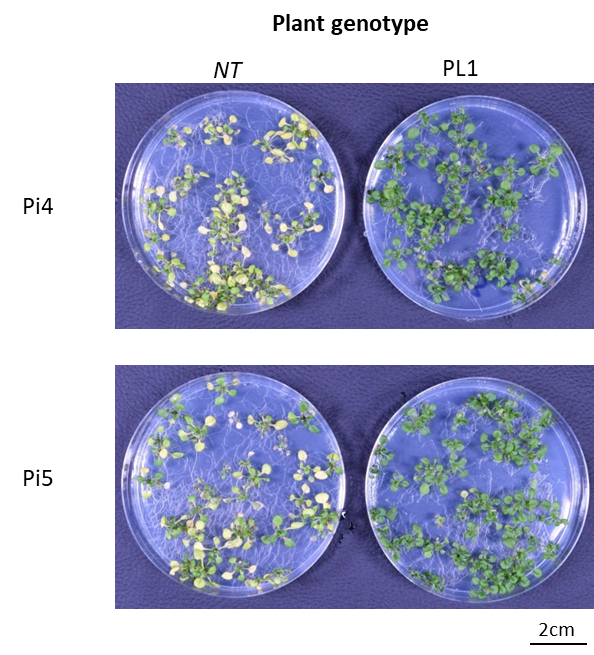
**

**Figure S14. PL1 insensitive strains of LMG5084 cannot induce disease symptoms in transgenic plants expressing PL1.** Fourteen-day-old non-transgenic (NT) and transgenic seedlings (PL1(1-2)) were flood inoculated with 1 x 10^6^ CFU mL^-1^ of PL1-insensitive *Ps* strains and symptoms were left to develop other 3 days. Pictures of plates and individual plants were taken 0- and 3-days post infection. Experiments were repeated 3 times with identical results.

| **Mutant strain** | **PL1 sensitivity** | **Predicted gene** | **Position in ORF (bp)** | **sequence 5’→3’ (WT v mutant)** |
| --- | --- | --- | --- | --- |
| Pi1 | >10 µM^a^ | Glycosyltransferase;  GenBank: CP005969.1 | 588 | AAGTTCTTTCTGTTCGTATTAC  AAGT---------------------------ATTAC |
| Pi2 | >10 µM^a^ | wbpL; glycosyltransferase;  GenBank: CP005969.1 | 333 | GCCTGGGCTTGT  GCCT--GGCTTGT |
| Pi3 | >10 µM^a^ | wpbM; nucleoside-diphosphate sugar epimerase;  GenBank: CP005969.1 | 285 | CTGCGGGAAACC  CTGCGG--AAACC |
| Pi4 | >10 µM^a^ | Phosphomannomutase;  GenBank: CP005969.1 | 1298 | CCGCGCCATCGGC  CCGCG**A**C**G**TCGGC |
| Pi5 | >10 µM^a^ | GDP-mannose 4,6-dehydrase;  GenBank: CP005969.1 | 763 | CGTGGCCGTGAG  CGTG--CCGTGAG |
| Pi6 | >10 µM^a^ | Glycosyltransferase;  GenBank: CP005969.1 | 647 | TACCCGGTGGTGATCCTCGGTGGCGGGC  TACC -----------------------------GGTGGCGGGC |
| Pi7 | >10 µM^a^ | wpbM  GenBank: CP005969.1 | 618 | GCGATGCAGT  GCGAT--CAGT |
| Pi8 | >10 µM^a^ | O-antigen ligase-like  GenBank: CP005969.1 | 606 | AGACGCGTACTGCACTGGT  AGACG------------------- --TGGT |

**Table S1. Mutations linked with PL1-resisistance.**

>10 µM^a^ – no clearing observed at >10 µM

| Primer name | Sequence 5’ -> 3’ |
| --- | --- |
| OPRF_F | AACTGAAAAACACCTTGGGC |
| OPRF_R | CCTGGGTTGTTGAAGTGGTA |
| ACT2_F | CTAAGCTCTCAAGATCAAAGGCTT |
| ACT2_R | ACTAAAACGCAAAACGAAAGCGGT |
| 18S_F | ATTGGAGGGCAAGTCTGGTGC |
| 18S_R | GCA GAA GGG ACG AGA CGA C |

**Table S2. qPCR primers used in this study**

| **Sanger ID** | **Sample** | **ENA accession number** |
| --- | --- | --- |
| 4526STDY7070045 | *Ps* 5084 Parent | SAMEA104233059 |
| 4526STDY7070060 | *Ps* 5084 Pi1 | SAMEA104233065 |
| 4526STDY7070076 | Ps 5084 Pi2 | SAMEA104233071 |
| 4526STDY7070084 | *Ps* 5084 Pi3 | SAMEA104233074 |
| 4526STDY7070092 | *Ps* 5084 Pi4 | SAMEA104233077 |
| 4526STDY7070100 | *Ps* 5084 Pi5 | SAMEA104233080 |
| 4526STDY7070108 | *Ps* 5084 Pi6 | SAMEA104233083 |
| 4526STDY7070116 | *Ps* 5084 Pi7 | SAMEA104233086 |
| 4526STDY7070037 | *Ps* 5084 Pi8 | SAMEA104233055 |

**Table S3. ENA accession numbers for sequenced samples**
